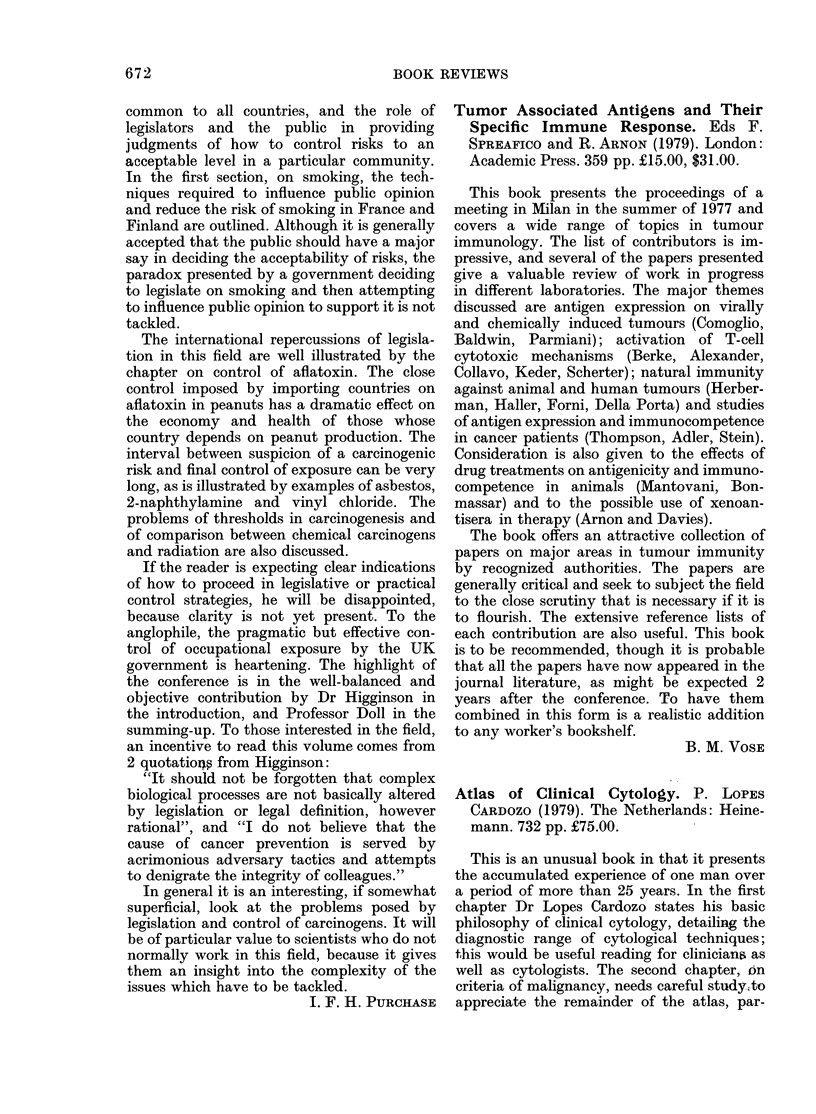# Tumor Associated Antigens and Their Specific Immune Response

**Published:** 1980-04

**Authors:** B. M. Vose


**Tumor Associated Antigens and Their Specific Immune Response.** Eds F. SPREAFICO and R. ARNON (1979). London: Academic Press. 359 pp. £15.00, $31.00.

This book presents the proceedings of a meeting in Milan in the summer of 1977 and covers a wide range of topics in tumour immunology. The list of contributors is impressive, and several of the papers presented give a valuable review of work in progress in different laboratories. The major themes discussed are antigen expression on virally and chemically induced tumours (Comoglio, Baldwin, Parmiani); activation of T-cell cytotoxic mechanisms (Berke, Alexander, Collavo, Keder, Scherter); natural immunity against animal and human tumours (Herberman, Haller, Forni, Della Porta) and studies of antigen expression and immunocompetence in cancer patients (Thompson, Adler, Stein). Consideration is also given to the effects of drug treatments on antigenicity and immunocompetence in animals (Mantovani, Bonmassar) and to the possible use of xenoantisera in therapy (Arnon and Davies).

The book offers an attractive collection of papers on major areas in tumour immunity by recognized authorities. The papers are generally critical and seek to subject the field to the close scrutiny that is necessary if it is to flourish. The extensive reference lists of each contribution are also useful. This book is to be recommended, though it is probable that all the papers have now appeared in the journal literature, as might be expected 2 years after the conference. To have them combined in this form is a realistic addition to any worker's bookshelf.

B. M. VOSE